# Effect of Dental Fear on Delay in Seeking Dental Treatment Among Adults in a South Asian Setting

**DOI:** 10.7759/cureus.87540

**Published:** 2025-07-08

**Authors:** Ribca Mariam, Jannat Faisal Malik, Muhammad Hammad Rafique, Azeem Hussain Soomro, Adonis Abu Kariem, Rima Jarbou, Ayesha Ibrar, Ibrahim Mahdi, Bassam Obied

**Affiliations:** 1 Dentistry, Shifa College of Dentistry, Islamabad, PAK; 2 Orthodontics, Lahore Medical and Dental College, Lahore, PAK; 3 Periodontics, Dow University of Health Sciences, Karachi, PAK; 4 Orthodontics, Dubai Health, Dubai, ARE; 5 Dentistry, Mohammed Bin Rashid University of Medicine and Health Sciences, Dubai, ARE; 6 Dentistry, Lahore Medical and Dental College, Lahore, PAK; 7 Dentistry, Segi University, Petaling Jaya, MYS

**Keywords:** dental anxiety, dental fear, dental insurance, dental neglect, socio-demographic factors, treatment delay

## Abstract

Background: Dental fear is a common reason why adults avoid the dentist, which can result in overlooked dental care and worsening oral health concerns. While new dental technology is available, a lot of people postpone treatments because they fear the thought of visiting the dentist. The purpose of this study is to examine why adults in Islamabad are slow to get dental treatment, looking at the effects of dental fear and factors such as age, gender, income, and dental insurance.

Methods: A cross-sectional study was done using data from 385 adults chosen from dental and community clinics. The tool selected for assessing dental anxiety was the Modified Dental Anxiety Scale (MDAS), and the Dental Neglect Scale (DNS) was used for deciding on treatment delays. Analysis of the data was done with descriptive statistics, chi-square tests, t-tests, ANOVA, Pearson correlation, and linear regression. Data collection took place from March to May 2025.

Results: A statistically significant yet limited relationship between dental anxiety and dental neglect was obtained (r = 0.129, p = 0.011; R 2 = 1.7%, approximately). Dental neglect showed a significant gender difference, with male participants giving significantly higher scores on neglect, but no significant difference was seen on the aspect of dental anxiety. Those who lack dental insurance are considerably more neglectful of their dental health. Regression analysis established that dental anxiety was a significant yet small predictor of dental neglect (B = 0.133, p = 0.011). Neglect and anxiety were also related to income and age, but with small to moderate effect sizes.

Conclusion: The findings indicate a weak relationship between dental anxiety and neglect, which implies that dental fear can be a potential factor that causes dental care delays among adults in Islamabad. Sociodemographic variables, including gender, income, and insurance status, also determined dental behavior. Future studies on such a population would require longitudinal assessment of causality, considering the small effect sizes due to the limitation of a cross-sectional design. However, specific measures can be provided, including patient education, anxiety adaptation measures, and increased access to preventive services to minimize the risk of dental care delay among vulnerable groups.

## Introduction

Dental fear is a common barrier preventing many adults from seeking timely dental care. Studies show that those with high dental anxiety tend to cruise through missed dental appointments or delay dental appointments. This neglect may worsen their situation [1]. Poor dental health and treatment of symptoms tend to become objects of concern. Thus, an apparent vicious cycle emerges whereby the higher the fear, the greater the avoidance of somatic disturbance and thus of anxiety, and the greater the dental problem [2].

Adults with dental fear avoid treatment for decades, and the fear usually starts in childhood because of traumatic histories. Drilling and anesthetics are primary triggers for fear, but empathetic behavior by the dentist can alleviate anxiety. Fear avoidance drastically creates poorer oral health, particularly among men [3]. World estimates indicate that dental fear affects approximately 15% of adults, with a greater prevalence among women and younger individuals [4]. Prevalence is impacted by the tools of measurement. It is critical to understand this common fear as a platform for the formulation of interventions to enhance dental care use and oral health outcomes [4].

Dental fear is like a clinical phobia and has cognitive, emotional, behavioral, and physiological features. While there are many self-report scales, few have sound theory-based orientations, resulting in ambiguity regarding how to define and measure dental fear and anxiety [5]. Extreme dental fear is experienced by about 16% of the population and is more prevalent in women and 40-64-year-old adults. The high-fear group delays dental attendance and has differences in socio-economic status and oral health compared to the low-fear group [6].

Dental anxiety is connected to intense psychosocial effects, especially in those experiencing multiple fears, and impacts psychological well-being, social interactions, and avoidance behaviors. Greater levels of anxiety were related to diminished morale and self-esteem, referencing the additional emotional toll of dental fear [7]. Dental anxiety is often tied to other fears, especially the fear of pain, which stands out as the biggest predictor for both men and women. Additionally, fears related to mutilation and claustrophobia play a role, particularly among women, indicating that dental fear frequently overlaps with more general patterns of fear [8].

It turns out that many patients put off getting treatment for dental care, mostly because of neglecting their dental health, trying to self-medicate, or simply not knowing better. Interestingly, factors like age or income didn’t really play a role in this delay, which points to issues related to behavior and a lack of awareness [9]. It's not uncommon for preschoolers to experience delays in dental treatment, often due to factors like fear of the dentist and a lack of understanding about oral health among parents. By focusing on these issues with specific interventions, we can potentially minimize these delays and enhance the dental health of young children [10].

According to the study, several challenges make it difficult for people to obtain prompt dental care. Among these reasons are money problems, bad memories from past dental appointments, lack of enough time, and stigma and discrimination in society. All of these points are important for deciding if people will be able to find and get the dental care they require [11]. It was found that about two-thirds of pediatric dental patients in Tanzania experienced delays before seeking dental care. It was mostly dental problems that caused the delays, instead of having to do with students’ social or economic status. Previous unpleasant experiences in dental care and wanting more attention to prevention made some people decide to postpone their visits [12]. Experts say that dental anxiety and not being confident about oral health affect delays in visiting the dentist more than merely procrastinating. Often, the high cost of treatment is seen as a big hurdle to getting help right away [13].

Improvements in dental management, instruction, and technology haven’t stopped the problem of dental anxiety in today’s public health. Seeing how this fear stops people from going to the dentist is important so that we can design successful strategies to reduce anxiety and promote early care, which can improve oral health. The goal is to understand the role of dental anxiety in making adults wait longer to visit the dentist. Examining the main reasons and the level of these delays is meant to give useful information to dentists, public health workers, and policymakers. In this way, they can create successful ways to handle this problem and help people receive dental care when needed.

Although the level of dental care has improved, a significant proportion of adults avoid or postpone treatment of dental problems because of fear and anxiety. Dental fear has continuously been linked to the avoidance of treatment, which may contribute to the deterioration of oral health, increased treatment expenses, and the creation of a requirement for invasive procedures. Oral health has also been related to poor health in a wider spectrum, such as nutritional deficiency, systemic ill health, as well as low quality of life. Strategies such as improved dentist-patient communication, environmental modifications, and behavioral support are believed to help reduce dental fear and improve adherence in some populations.

There is a lack of public health data in the Pakistani context, and specifically in urban regions, such as Islamabad on the overlap between dental anxiety and neglect. This exploration seeks to address this gap by investigating the prevalence, aetiology, and demographic correlates of dental fear and its relation to delayed dental visits. The results can be used in clinical practice and community-based health promotion programs to enhance the status of oral health and decrease the tendency to avoid treatment because of fear.

The primary objective of the study is to investigate the association between dental anxiety and delayed/avoided dental treatment in the adult population of Islamabad, Pakistan, through the verified psychometric scales.

Secondary objectives are to assess the prevalence of dental fear and anxiety in the study population, determine factors that are commonly self-reported as dental fear and avoidance, examine how dental anxiety and dental neglect are affected by the sociodemographic factors (age, gender, level of education, and income), and investigate the moderating role of the presence or absence of dental insurance in the connection between anxiety and care-seeking behavior.

## Materials and methods

Study design and methods

The purpose of this study was to investigate the effect of dental fear on delays in seeking dental care among adults, using a cross-sectional design. Data were collected in Islamabad, Pakistan, from individuals in need of dental treatment. Participants were recruited from a combination of public dental outpatient departments, private practices, and community health centers. These recruitment sites included university-affiliated clinics and women-focused community clinics, which primarily serve young adults, low-income individuals, and widowed or divorced women. This approach allowed for a diverse sample in terms of age, gender, education, and socioeconomic status, although certain groups, such as younger women and individuals without a partner, were overrepresented. Standardized and validated questionnaires were used to assess dental anxiety and neglect, supplemented by interview-based items exploring reasons for dental avoidance. Details such as age, gender, education level, and health were collected to see if they could influence dental anxiety and whether patients received dental care. Due to the study’s design, the study could explore the relationship between dental fear and late dental care, pointing out the primary reasons many adults do not seek timely dental treatment.

Sample size and technique

A formula for an infinite population was used to calculate the sample size since that was the only way to know how many adults feel dental fear in the population. The way to calculate is with this formula:

\[n = \frac{Z^2 \cdot p (1 - p)}{d^2}\]

Where Z in this formula refers to the standard score that matches the required level of confidence, p is the estimated proportion, and d is the pardonable margin of error. Standard level of trust (95%) was applied (Z = 1.96), whereas d was determined at 0.05. The likelihood of a false-negative (p) was set to 0.50 to give the largest possible sample size required [14].

Convenience sampling was used to recruit participants, which included the public and private dental clinics and community health centers in Islamabad. The adult patients who went to these sites and met the inclusion criteria were invited to get involved. Patients waiting at the clinic were approached by trained data collectors who informed them about the study's purpose and sought their consent. Of the 412 people who were approached, 385 accepted to participate, leading to a response rate of 93.4%. There were no incentives to participate.

The eligibility of study participants was determined based on specific inclusion and exclusion criteria, as detailed in Table 1.

**Table 1:** Inclusion and Exclusion Criteria for Study Participants

Inclusion Criteria	Exclusion Criteria
Adults who are 18 years old and older	Anyone younger than 18 years
Individuals who have postponed dental treatment for at least a month	People who have never been to a dentist (no prior treatment)
Those who can and are willing to give informed consent	Individuals with diagnosed mental health issues that affect their perception of fear or anxiety
Patients visiting dental clinics (whether public or private) for treatment or consultation	Patients needing emergency dental care at the time of data collection
Individuals who can understand and respond to the questionnaire in the local language	People with cognitive or communication challenges that make it difficult to complete the questionnaire

Data collection tools

To gather information, we prepared a questionnaire with three essential sections: demographics, a study on dental fear, anxiety, and treatment preferences. The researchers used some standardized tests, as well as questions made by themselves for the questionnaire.

Demographic information

Initially, participants were asked for their demographic information in order to learn about their backgrounds. Age, gender, marital status, level of education, the job, and how often dental appointments are kept were considered essential variables. By studying these factors, it was possible to assess whether they could contribute to dental fear or a delay in getting dental care. Exploring the characteristics that define the study population gave a clear idea about why there are differences in fear and anxiety levels, which helped analyze how these emotions affect dental care-seeking.

Modified Dental Anxiety Scale (MDAS)

We assessed the participants’ dental anxiety in the second section using the MDAS. The MDAS consists of just five questions that help to diagnose dental anxiety by exploring everyday dental situations such as seeing the dentist, waiting for treatment, and getting anesthesia. Questions are given scores of 1 to 5, where one is not anxious and five is very nervous, providing a possible total score between 5 and 25. A result of 19 or higher indicates that dental care is significant for the person. Because of this scale, we could track how much anxiety different participants had, which allowed us to find out who could be avoiding dental care because of their anxieties. Humphris, Morrison, and Lindsay created the MDAS in 1995, and many studies show that its excellent reliability across different groups was proven by a range of 0.83 to 0.92 for Cronbach’s alpha coefficients [15]. We requested the original authors for formal approval before we decided to use the MDAS in this study. Though the MDAS is a validated scale, it was used in the original English version, and it has not been revalidated in Pakistani people in terms of culture and language.

Dental Neglect Scale (DNS)

The DNS was applied here to assess how much attention participants give to their oral health. This scale was introduced in 1992 by Newton, Bower, and Williams and consists of six items that meet all the validity rules. The items check for various aspects, for example, how regularly a person practices oral care, goes to the dentist, and is responsible for their dental health. Every item on the questionnaire is given a score from 1 (strongly disagree) to 5 (strongly agree), and a higher score indicates there is more dental neglect. DNS scores are very similar across its items, as Cronbach’s alpha values are between 0.70 and 0.85, meaning it is a reliable tool for studies [16]. We obtained official authorization from the original authors to make sure everything was done ethically and properly using the scale. The DNS was also administered in its initial form in English in this research without swerving in the local context.

Procedure

After providing informed consent, participants were recruited through local dental outpatient departments, public dental clinics, and through local dental practices. Self-administered surveys were conducted via paper-based forms in English. Some were able to fill in the questionnaires on their own, and there were those who were helped to fill them by the trained data collectors with the aid of standardized and neutral encouragers. No personal identifying data were taken, and the answers were coded with numbers and made anonymous. The respondents were surveyed in March to May 2025, where a large enough sample and the representative adult population of the clinic-goers in Islamabad could be accessed. Treatment delay was defined as a one-month delayed dental visit or more, even when the respondent knew that they needed dental treatment. The measure was based on the question: Have you postponed a dentist-advised appointment of over a month due to fear, budget, or any other factor? The responses to this item were analyzed to identify patterns of avoidant behavior regarding dental care among participants.

Statistical analysis

The data was entered and analyzed using IBM SPSS Statistics for Windows, Version 26 (Released 2019; IBM Corp., Armonk, New York, United States). The data was analyzed with both descriptive and inferential statistical methods. The data on each participant’s demographics and medical background were summarized with descriptive statistics and frequencies. Inferential statistics were applied to explore how study variables influence each other and to show the differences. Pearson correlation was used to study the connection between the MDAS and the DNS. The independent samples t-test method was used to find out if any differences in scores were present between MDAS and DNS for men, women, and those with or without dental insurance. A one-way ANOVA test was conducted to check if there are significant differences in age groups and income groups. Linear regression was carried out to find out how likely MDAS scores are to predict DNS scores. Also, chi-square tests were performed to see the relationships between categorical variables such as age, gender, and marital status. The value chosen for the significance level was p < 0.05. They proved helpful in finding all the details and relationships between the main elements in the study.

Ethical considerations

The study was carried out according to ethical principles for research with humans. Before data collection began, the study protocol was checked and approved by the Institutional Review Board (IRB) of the NeuroWave Research Center (IRB-2025-0028). The ethical review made sure that everyone participating was safe and their privacy and rights were respected during the research. All participants were informed and gave consent before participation. Participants were given all the information they needed about the study’s objectives, the procedures to be followed, and what could happen as a result. People chose to take part themselves, aware that they could leave the study at any stage by choice.

All data were kept secure by giving every questionnaire its own code. No personal details were asked for or shown. Also, both the MDAS and DNS were used, and the researcher obtained permission from their developers to include them properly in the study according to licensing.

## Results

Table 2 presents the demographic characteristics of the study participants. The largest age group was 18-25 years (n = 171, 44%), followed by 36-45 years (n = 88, 23%). Adults aged 55 years and above were underrepresented (n = 8, 2%), likely reflecting the younger patient population served by the university dental clinics included in the study. In terms of gender, the majority of participants were female (n = 262, 68%), while male participants were 123 (32%). This imbalance may be attributed to the inclusion of community dental clinics that primarily serve women, including widows and low-income women.

**Table 2:** Demographic Characteristics of Participants (N=385) f: frequency, %: percentage

Variable	Category	f	%
Age	18–25 Years	171	44
	26–35 Years	59	15
	36–45 Years	88	23
	46–55 Years	59	15
	55 Years or Above	8	2
Gender	Male	123	32
	Female	262	68
Marital Status	Single	71	18
	Married	83	22
	Divorced/Separated	136	35
	Widowed	95	25
Educational Level	No Formal Education	48	12
	Primary School	94	24
	Secondary/High school	132	34
	Bachelor’s Degree	80	21
	Master’s Degree or Above	31	8
Occupation	Student	51	13
	Employed	61	16
	Self-employed	110	29
	Unemployed	89	23
	Retired	74	19
Monthly Income	Less than 30,000 PKR	62	16
	30,000–60,000 PKR	67	17
	60,000–100,000 PKR	75	19
	More than 100,000 PKR	93	24
	Prefer Not to Say	88	23
Dental Insurance Coverage	Yes	178	46
	No	207	54
Dental Visit Frequency	Every six months	57	15
	Once a Year	80	21
	Only When in Pain	171	44
	Rarely/Never	77	20
Postponed Dental Visit Due to Fear	Yes	101	26
	No	155	40
	Maybe	129	34
Negative Dental Experience in the Past	Yes	125	32
	No	166	43
	Maybe	94	25

Concerning the marital status, the most significant number was divorced or separated individuals (n = 136, 35%), followed by the widowed participants (n = 95, 25%). Smaller proportions comprised married participants (n = 83, 22%) and single persons (n = 71, 18%). This distribution likely reflects the patient demographics of public and community dental clinics offering subsidized services to underserved female populations. As for education, the largest segment has secondary or high school education (N=132, 34%), with 24% (N=94) having completed primary school, and only 8% (N=31) holding a master’s degree or higher. In the workforce, self-employed individuals are the largest group at 29% (N=110), followed by the unemployed at 23% (N=89) and retirees at 19% (N=74). As for monthly income, the highest proportion of participants reported earning more than 100,000 PKR (n = 93, 24%), while a substantial number (n = 88, 23%) chose not to disclose their income, possibly due to cultural or privacy-related concerns. As for dental visits, a significant portion only go to the dentist when they’re in pain (N=171, 44%), while 21% (N=80) visit once a year, and 15% (N=57) go every six months. Interestingly, 20% (N=77) rarely or never see a dentist. Anxiety about dental visits is also a factor, with 26% (N=101) admitting to avoiding appointments, while 40% (N=155) have not, and 34% (N=129) are unsure. Lastly, when asked about negative or painful dental experiences, 32% (N=125) reported having such experiences, 43% (N=166) said they haven’t, and 25% (N=94) are uncertain.

Table 3 presents the relationship between the two primary study variables: the MDAS and the DNS. The results indicate a statistically significant but weak positive correlation (r = 0.129, p < 0.05) between dental anxiety and dental neglect. This finding suggests that higher levels of dental anxiety are modestly associated with increased dental neglect. Although the strength of the correlation is limited, the association remains statistically significant. Both variables were significantly correlated at the 0.05 level, as indicated by the asterisk.

**Table 3:** Intercorrelation between the Study Variables *p <0.05 considered statistically significant; r: Pearson Correlation

Variable	r	p
The Modified Dental Anxiety Scale & Dental Neglect Scale	0.129	0.011^*^

Table 4 shows the differences in the scores of MDAS and DNS between male participants and female participants. In case of MDAS, the female group (M = 13.93, SD = 2.22) rated slightly higher than the male group (M = 13.84, SD = 2.69). This difference was not insignificant (t = -2.45, p = 0.015); nevertheless, the effect size was also not large (Cohen d = 0.38); there could be the implication that the above difference is not a clinically significant one. Conversely, the DNS scores of the male participants (M = 18.62, SD = 2.57) were much higher as compared to those of female participants (M = 18.23, SD = 2.39) and the difference was statistically significant (t = 2.80, p = 0.006) though the effect size, in this case, is smaller (Cohen d = 0.42). It is an indication that there is a small to medium effect on the practical differences in dental neglect between genders.

**Table 4:** Comparison among Variables (Gender) M: mean; SD: standard deviation; LL: lower limit; UL: upper limit; Cl: confidence interval; Independent t-test, *p < 0.05 considered statistically significant.

Variable	Male (N=123); M±S.D	Female (N=262); M±S.D	t	p	Cl 95% LL	UL	Cohen’s D
The Modified Dental Anxiety Scale	13.84±2.69	13.93±2.22	-2.45	0.015^*^	-0.65	-0.07	0.38
Dental Neglect Scale	18.62±2.57	18.23±2.39	2.80	0.006^*^	0.12	0.68	0.42

Table 5 provides a comparison of the MDAS and the DNS for individuals with dental insurance (N=178) versus those without (N=207). For the MDAS, those with insurance have an average score of 13.84 (SD=2.32), while those without insurance score a bit higher at 13.95 (SD=2.43). The independent t-test revealed a statistically significant difference in the MDAS scores between participants with and without dental insurance (t = -0.448, p = 0.006); however, the effect size was negligible (Cohen’s d = 0.046), indicating that the observed difference is unlikely to be clinically meaningful. For the DNS, participants with insurance reported a mean score of 18.21 (SD = 2.52), compared to 18.48 (SD = 2.39) for those without insurance. The t-test also indicated a statistically significant difference (t = -1.100, p = 0.007), with a small effect size (Cohen’s d = 0.110), suggesting a slight increase in dental neglect behaviors among those without insurance. The 95% confidence intervals suggest that the magnitude of the difference between groups is small.

**Table 5:** Comparison among Variables (Dental Insurance Coverage) M: mean; SD: standard deviation; LL: lower limit; UL: upper limit; Cl: confidence interval; Independent t-test, **p <0.05, *p <0.01 considered significant.

Variable	Yes (N=178); M±S.D	No (N=207); M±S.D	t	P	Cl 95% LL	UL	Cohen’s D
The Modified Dental Anxiety Scale	13.84±2.32	13.95±2.43	-0.448	0.006^**^	-0.587	0.369	0.046
Dental Neglect Scale	18.21±2.52	18.48±2.39	-1.100	0.007^**^	-0.767	0.217	0.110

Table 6 presents a comparison of MDAS and DNS scores across five age groups. For the MDAS, the mean scores showed slight variation, with the lowest average in the 18-25 years group (M = 13.67, SD = 1.94) and the highest in the 36-45 years group (M = 14.25, SD = 2.58). A one-way ANOVA revealed a statistically significant difference among the age groups (p = 0.003), though the effect size was small (η² = 0.012), indicating that age had a minimal impact on dental anxiety levels.

**Table 6:** Comparison of Variables (Age) M: mean; S. D: standard deviation; F: F-ratio; η2: effect size; One-way ANOVA, **p <0.05, * p <0.01 considered significant.

Variable	18-25 years (N=171); M±S.D	26-35 years (N=59); M±S.D	36-45 years (N=88); M±S.D	46-55 years (N=59); M±S.D	55 years or above (N=8); M±S.D	p	F (4,380)	η2
The Modified Dental Anxiety Scale	13.67±1.94	14.15±2.68	14.25±2.58	13.78±2.77	14.13±3.04	0.003^**^	1.113	0.012
Dental Neglect Scale	17.72±2.11	18.22±2.49	19.15±2.61	19.20±2.41	18.00±3.38	0.023^*^	7.524	0.073

In contrast, DNS scores demonstrated a clearer trend, with mean values increasing with age, peaking in the 46-55 years group (M = 19.20, SD = 2.41). The ANOVA for DNS also indicated a statistically significant difference (p = 0.023) and a moderate effect size (η² = 0.073), suggesting that age had a more meaningful impact on dental neglect behaviors compared to anxiety. The findings indicate that whereas age is statistically linked to dental anxiety and neglect, its practical effect remains greater in neglect, particularly in older age groups.

Table 7 demonstrates how the MDAS and the DNS differ about how much monthly income people make. Among those earning between 30,000 and 60,000 PKR, the average MDAS score was 13.67 (SD = 2.29), whereas individuals earning less than 30,000 PKR had a higher average score of 14.15 (SD = 2.27). A statistically significant difference was found (p = 0.006), although the effect size was very small (η² = 0.004), indicating only a minimal relationship between income and dental anxiety. Interestingly, despite reporting higher anxiety, participants in the lowest income group also reported more frequent dental visits. This apparent contradiction may be explained by the likelihood that low-income individuals primarily seek dental care for symptomatic or urgent needs (e.g., pain or infection) rather than preventive checkups. As for the DNS, people earning less than 30,000 PKR have an average of 17.44 (SD = 1.87), while those earning more than 100,000 PKR have an average of 18.84 (SD = 2.25). According to the ANOVA, there is a significant difference (p = 0.005) and a noticeable effect size (η² = 0.039), which indicates that monthly income has a stronger influence on dental neglect. The effect size falls within the small range, suggesting a modest but meaningful impact. It is important to note that 23% of the respondents chose the category Prefer not to say in the case of income, and the latter were included in the ANOVA. However, this is not a classification of a genuine income bracket, and inclusion may have restricted interpretations of income effects. When analyzing in the future, this group should be considered to be excluded, wherein a more accurate comparison within subgroups can be made.

**Table 7:** Comparison of Variables (Monthly Income) M: mean; S. D: standard deviation; F: F-ratio; η2: effect size; One-way ANOVA, ** p <0.05, *p <0.01 considered significant.

Variable	Less than 30,000 PKR (N=62); M±S.D	30,000-60,000 PKR (N=67); M±S.D	60,000-100,000 PKR (N=75); M±S.D	More than 100,000 PKR (N=93); M±S.D	Prefer not to say (N=88); M±S.D	p	F (4,380)	η2
The Modified Dental Anxiety Scale	14.15±2.27	13.67±2.29	13.97±2.77	13.83±2.09	13.92±2.47	0.006^**^	0.358	0.004
Dental Neglect Scale	17.44±1.87	18.69±2.35	18.52±2.72	18.84±2.25	18.10±2.67	0.005^**^	3.830	0.039

Table 8 illustrates linear regression results where DNS scores are predicted with respect to the MDAS scores. The model was statistically significant (F(1,383) = 6.495, p = 0.011); however, it explained a small amount of variance (R 2 = 0.017; Adjusted R 2 = 0.014). The regression coefficient of the MDAS was B = 0.13 (95% CI: 0.03 to 0.24), which shows that when dental anxiety increased by one point, dental neglect also increased by 0.13 points. The effect size was low (0.129), which implies that although anxiety is statistically a predictor of neglect, it does not explain a large magnitude of variance in the neglect scores.

**Table 8:** Linear Regression Analysis Predicting Dental Neglect Scale (DNS) Scores using the Modified Dental Anxiety Scale (MDAS) B: coefficient; S. E: standard error; β: standardized coefficient; LL: lower limit; UL: upper limit; Cl: confidence interval, *p < 0.05, **p < 0.001 considered statistically significant.

Variable	B	95% Cl LL	UL	S.E	β	R^2^	Adjusted R^2^	F (1,383)	P
Model Summary	-	-	-	-	-	0.017	0.014	6.495	
Constant	16.51	15.06	17.95	0.74	-	-	-	-	<0.001^**^
The Modified Dental Anxiety Scale	0.13	0.03	0.24	0.05	0.129	-	-	-	0.011^*^

**Figure 1:**
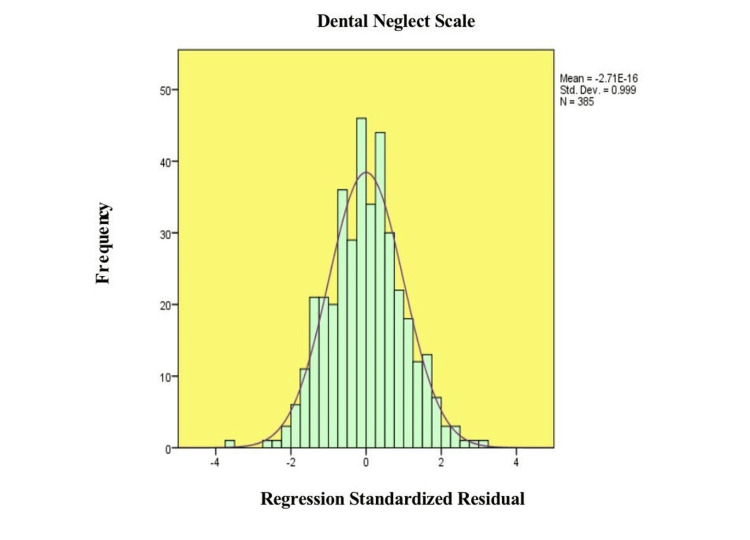
Histogram of Regression Standardized Residuals for the Dental Neglect Scale

Figure 1 represents a histogram with a normal distribution curve overlaid on it, which is the distribution of the standardized residuals of the regression model. The bell-shaped distribution is centered around a mean of approximately zero (-2.71 × 10⁻¹⁶), with a standard deviation close to 1 (SD = 0.999), indicating conformity to the assumption of normality. There is little skewness or kurtosis which is depicted by the bars of the histogram being very close together with the overlaid curve. These findings show that the residuals follow a normal distribution implying that the linear regression model is valid.

Table 9 shows the correlation between age groups and two factors, which are the fear of visiting the dentist and how often they visit the dentist. There was a statistically significant association between age and dental fear (21.39, df = 8, p = 0.038), and it showed that the level of fear is distributed in different age groups. The highest measures of anxiety were amongst the 18-25 age group ("Yes" = 50, "Maybe" = 70). Moreover, the frequency of visits to dentists was strongly related to the age variable (61.6, df 12, p < 0.001). The participants, especially those in the 18-25 age bracket, tended to make regular check-ups, such as every six months or annually, whereas participants aged 55 and above tended to make occasional visits when in pain or hardly ever. These findings suggest that younger individuals are more likely to maintain regular dental visits, despite reporting higher levels of anxiety.

**Table 9:** Descriptive Statistics of Demographic Variables (Age, Fear or Anxiety of Dental Visit, and Visit to Dentist) f: frequency; %: percentage; df: degree of freedom; x2: effect size; p: level of significance; p-values calculated using the chi-square test; **p < 0.05, **p < 0.001 considered statistically significant.

Variables	f	Yes	Fear of dental visit No	May be	df	p	x^2^	Every 6 months	Once a year	Visit to a dentist Only when in pain	Rarely/never	df	p	x^2^
Age	-	-	-	-	8	0.038^*^	16.3	-	-	-	-	12	<0.001^**^	41.6
18-25 years	171	50	51	70	-	-	-	38	38	60	35	-	-	-
26-35 years	59	14	25	20	-	-	-	5	17	30	7	-	-	-
36-45 years	88	21	44	23	-	-	-	6	9	53	20	-	-	-
46-55 years	59	14	31	14	-	-	-	5	12	28	14	-	-	-
55 years or above	8	2	4	2	-	-	-	3	4	0	1	-	-	-

Table 10 shows the correlation between the monthly income levels and the other two factors, such as dental fear and the frequency of dental visits. Income was also statistically related with fear of dental visits (x 2 = 9.12, df = 8, p = 0.033), although the fear was recorded in all income brackets, it was highest among the 30,000-60,000 PKR bracket and the PKR >100,000 bracket (accounting 24 yes responses, respectively).

**Table 10:** Descriptive Statistics of Demographic Variables (Monthly Income, Fear or Anxiety of Dental Visit, Visit to Dentist) f: frequency; %: percentage; df: degree of freedom; x2: effect size; p: level of significance; p-values calculated using the chi-square test; *p < 0.05 considered statistically significant, the significance level is set at p < 0.05.

Variables	f	Yes	Fear of dental visit No	May be	df	p	x^2^	Every 6 months	Once a year	Visit to dentist Only when in pain	Rarely/never	df	p	x^2^
Monthly income	-	-	-	-	8	0.033^*^	9.12	-	-	-	-	12	0.011^*^	25.9
less than 30,000 PKR	62	15	23	24	-	-	-	17	11	21	13	-	-	-
30,000-60,000 PKR	67	24	30	13	-	-	-	7	9	36	15	-	-	-
60,000-100,000 PKR	75	17	33	25	-	-	-	5	17	41	12	-	-	-
more than 100,000 PKR	93	24	35	34	-	-	-	12	19	37	25	-	-	-
prefer not to say	88	21	34	33	-	-	-	16	24	36	12	-	-	-

Also, income had an important relationship with the frequency of dental visits (X 2 = 25.9, df = 12, p = 0.011). Also, income had an important relationship with the frequency of dental visits (X 2 = 25.9, df = 12, p = 0.011). Interestingly, people having the lowest income bracket (<30,000 PKR) have shown the maximum frequency of regular six-month dental visits (n = 17) compared to the people having the 60,000-100,000 PKR income bracket, who are more likely to visit a dentist when they are feeling uncomfortable (n = 41). These results support a significant association between monthly income and both dental anxiety and visit frequency, highlighting socioeconomic disparities in oral healthcare behavior.

## Discussion

This study looks at the link between fear of the dentist and difficulties in getting dental care for adults in Islamabad, Pakistan. According to our findings, dental anxiety is strongly linked with dental neglect, suggesting that having more anxiety increases the likelihood of neglecting dental care. The results fit with those of previous studies, demonstrating that dental anxiety acts as a mediator connecting mindfulness, self-compassion, and dental neglect. Based on these findings, certain psychological factors may lead to avoidant dental habits [17].

We did not find any significant gender variation in dental anxiety, and the effect size was small. This is consistent with earlier studies, which indicated that dental anxiety had no association with gender, age, or education but showed a substantial relationship between prior traumatic dental experience and elevations in anxiety. This confirms the idea that individualized dental history can be a stronger concept than demographic features [18]. We found that male participants had higher dental neglect scores than female participants, although this gender effect is small (Cohen's d = 0.159). This is compatible with the past studies, which also reported an increased percentage of dental neglect in men. Such findings imply that the gender-related behavior patterns might play a role in oral health practices, regardless of the effect size being small [16].

In a study of ours, we also found that people without dental insurance had slightly higher dental anxiety scores, but the effect size was negligible. This is in line with the previous reports that there is a relationship between dental anxiety and poor attendance at dental clinics, especially in those who are covered by health insurance. These findings indicate that anxiety might exist in all groups, but its effects on conduct occur depending upon the accessibility and affordability [19]. In our study, individuals who did not have dental insurance had significant dental neglect, and the effect size is small. This goes in line with prior evidence that insurance status is significantly linked to frequent visits to the dentist, meaning that structural access barriers like the absence of insurance may also have a role in the malpractice of oral health practices [19]. Taken together, the combined influence of low insurance coverage and high out-of-pocket costs underscores affordability as a pivotal driver of dental neglect in Pakistani adults.

Our research analysis revealed that age was a statistically significant but weak predictor of dental anxiety (d2 = 0.012), and the practical outcome was minimal. It is generally consistent with the previous studies that also revealed an age difference in anxiety, but failed to indicate its effect size. Together, these findings suggest that while age contributes to dental anxiety, its impact is modest compared with other factors such as past dental experience or perceived treatment severity. [20]. We found that both age and the degree of dental neglect were correlated, where older age groups have higher DNS scores. This was a statistically significant moderate association (0.073), which showed a meaningful relationship. This finding is also supported by previous research, which reveals that oral health problems and neglect are more common among the elderly because of caregiver responsibilities, a reduced ability to care for themselves, and obstacles in the system (like inadequate hygiene, unchecked problems, or delays in diagnosis and treatment) [21].

Participants with lower income exhibited a higher rate of dental anxiety, but the effect was small (eta squared = 0.004) in our study. This correlates with other studies that refer to low income as a major institutional factor that leads to dental anxiety, in addition to other issues like the dissatisfaction component of oral health and check-ups. The results are essential to emphasize the complex exposure of already economically disadvantaged populations to anxiety and lack of engagement in care [22]. In our study, there was a significant result with a small to moderate effect size that lower-income participants had a significantly greater score of dental neglect. This result is consistent with a population-based study, which also revealed financial disparities in dental neglect. These findings show the potential impact of financial constraints on access to professional dental care by individuals [23].

We found that dental anxiety shared a statistically significant relationship with dental neglect (B = 0.133, 8 = 0.129, p = 0.011), but the effect size was small. This implies that although anxiety may be one of the causes of negligent dental practices, it probably occurs in combination with other influencing factors like access, motivation, and past experiences. In line with this, past studies have established the role of dental anxiety as a mediator between psychological factors like mindfulness and dental neglect and established the involvement of psychology in avoidant behaviors. These findings together indicate that even modest levels of anxiety may influence oral health outcomes, reinforcing the value of early psychological screening and intervention [17].

In our research, dental anxiety was most significant amongst younger adults (18-25 years); however, the overall influence of age was minimal (eta 2 = 0.012). This concurs with previous studies where it has been found that the majority of dental anxiety originated when one was a child or during the adolescence stage, due to a bad experience or even family influence. This may be the reason why such visits show increased anxiety among the younger groups, even though they appear to visit more often. Such findings highlight the implementation of early dental interventions to eliminate the development of avoidance behaviors in the long term [24]. In our study, younger participants were more likely to regularly visit a dentist, whereas older people sought treatment when they needed it due to pain. This trend is consistent with the results of previous studies, as aging affects the frequency of dental visits, with younger people having a higher rate of preventive care [19].

In our study, we found that dental fear was prevalent within the lower-income group as well as within the mid- and high-income populations. This is in line with previous evidence, in which dental anxiety was greater in less deprived individuals, indicating that the presence of psychological issues might remain despite a different socioeconomic standing. The outcomes question the idea that anxiety only occurs among disadvantaged populations and necessitate the necessity of anti-anxiety measures on all income classes [25]. According to our results, poorer participants had higher facility rates of routine dental visits, whereas medium- and high-income populations tended to postpone their care until some pain had developed. This is opposed to conventional beliefs and is in line with a study conducted previously, where income mediated the effects of insurance on the frequency of visits, demonstrating the intricate nature of socioeconomic factors on dental care behavior. A reason may be that people with low incomes in our environment have access to subsidized or government-supported dentist clinics that encourage checkups compared to high-income households, as they will put off dental care because of financial costs, time constraints, or low perceived necessity [26].

These results suggest larger disparities in socioeconomic groups in Pakistan, relative inaccessibility of affordable dental care, and societal underdevelopment of oral health service systems. Low insurance coverage, out-of-pocket spending, and insufficient oral health education are likely to exacerbate the problem of delayed treatment and avoidance due to fear. Furthermore, even though women were adequately included in our sample, there is a possibility that the sociocultural norms in Pakistan extend into the health-seeking behavior of women and older adults, especially in low-income or rural families.

Limitations

Although the study provides valuable information about the connection between dental fear and treatment delays, there are various limitations that need to be mentioned. First, the cross-sectional design does not allow one to establish causality. Even though the results suggest that there is a statistically significant association between anxiety and dental neglect, it is not clear whether neglect is caused by anxiety or vice versa, or whether neglect increases anxiousness. Second, there is a selection bias given that there was the use of convenience sampling, especially at university and community dental clinics. The given method underestimates the people with the highest level of dental fear who do not visit dentists at all, and it is ironically the most interesting group. Consequently, the findings are mainly an indication of the willing participants who will commit to visiting dental clinics despite their anxiety. Third, the sample has demographic imbalances such as overrepresentation of women, young adults, and those who are unmarried. All this narrows down the applicability of the results to not only the general population of Islamabad but to parts of other areas that have different socioeconomic or cultural issues as well. Lastly, the self-reports are prone to recall bias and social desirability that can affect the truthfulness of the answers concerning anxiety and oral health behavior. Although the MDAS and DNS are validated instruments, this study failed to revalidate them in a local Pakistani population, and this might affect the cultural sensitivity of some items.

Future directions

Since this study is a cross-sectional study, longitudinal studies are required in the future to have a better handle on the causality of the relationship between dental anxiety and neglect. Prospective cohort studies may assist in answering the question of whether early interventions introduced to prevent fear led to ultimately positive changes in behavior related to dental care. It would also be worth investigating the groups of people who do not attend dental clinics at the moment, because they could have a greater rate of fear and neglect than is reflected here. The application of cognitive-behavioral therapy, environmental modifications, and digital techniques such as virtual reality appears to be promising in the treatment of anxiety. Still, the changes the techniques bring to actual dental practice have not been fully understood yet. The investigation of mutual relations between dental fear and comorbid disorders, including generalized anxiety disorder or depressive mood, could help learn more about more effective and individual approaches to treatment as well.

## Conclusions

This study highlights a statistically significant, though modest, association between dental anxiety and dental neglect among adults in Islamabad. Sociodemographic variables such as age, gender, and income were also found to influence these behaviors to varying degrees. While causality cannot be established due to the cross-sectional design, the findings suggest that dental fear may contribute to delayed care-seeking. Future efforts could focus on awareness campaigns, improved dentist-patient communication, and low-barrier access to preventive services. However, given the limitations of the study, including sampling bias and limited generalizability, further longitudinal and population-based research is needed to inform effective interventions.

## Appendices

**Table 11:** Demographic Information Questionnaire

Items	Responses
Age	-
Gender	-
Educational Level	-
Occupation	-
Monthly Income (pkr)	-
Marital Status	-
Do you have dental insurance coverage?	-
How often do you visit the dentist?	-
Have you ever postponed or avoided a dental visit due to fear or anxiety?	-
Have you ever had a negative or painful experience during a dental visit in the past?	-
